# Cytotoxic Constituents from the Sclerotia of *Poria cocos* against Human Lung Adenocarcinoma Cells by Inducing Mitochondrial Apoptosis

**DOI:** 10.3390/cells7090116

**Published:** 2018-08-24

**Authors:** Seulah Lee, Seul Lee, Hyun-Soo Roh, Seong-Soo Song, Rhim Ryoo, Changhyun Pang, Kwan-Hyuck Baek, Ki Hyun Kim

**Affiliations:** 1 School of Pharmacy, Sungkyunkwan University, Suwon 16419, Korea; sarahlee0801@gmail.com; 2 Department of Molecular and Cellular Biology, Samsung Medical Center, Sungkyunkwan University School of Medicine, Suwon 16419, Korea; leeseul6988@naver.com (S.L.); hyunsno@naver.com (H.-S.R.); songss911303@gmail.com (S.-S.S.); 3 Special Forest Products Division, Forest Bioresources Department, National Institute of Forest Science, Suwon 16631, Korea; rryoo@korea.kr; 4 School of Chemical Engineering, Sungkyunkwan University, Suwon 16419, Korea; chpang@skku.edu

**Keywords:** *Poria cocos*, polyporaceae, triterpenoid, sterol, diterpenoid, lung cancer, cytotoxicity, apoptosis

## Abstract

Previous studies have revealed the antitumor potential of *Poria cocos* Wolf against a broad spectrum of cancers. However, the biological activity of *P. cocos* against lung cancer, which is known as the leading cause of cancer mortality worldwide, and its underlying chemical and molecular basis, remain to be investigated. We aimed to evaluate the in vitro cytotoxicity of *P. cocos* toward human lung adenocarcinoma cells with different *p53* statuses, to identify the bioactive constituents of *P. cocos*, and explicate the molecular mechanisms underlying the cytotoxicity of these constituents in human lung adenocarcinoma cells. An EtOH extract of the sclerotia of *P. cocos* exhibited cytotoxicity toward four human lung cancer cell lines: A549, H1264, H1299, and Calu-6, regardless of their *p53* status. Chemical investigation of the extract resulted in the isolation of two triterpenoids, dehydroeburicoic acid monoacetate (**1**) and acetyl eburicoic acid (**4**); a sterol, 9,11-dehydroergosterol peroxide (**2**); and a diterpenoid, dehydroabietic acid (**3**). All of the isolated compounds were cytotoxic to the lung adenocarcinoma cell lines, exhibiting IC_50_ values ranging from 63.6 μM to 171.0 μM at 48 h of treatment. The cytotoxicity of the extract and the isolated compounds were found to be mediated by apoptosis, and accompanied by elevated Bax expression and/or Bcl-2 phosphorylation along with caspase-3 activation. Our data demonstrate that the sclerotium of *P. cocos* and its four bioactive constituents (**1**–**4**) exert cytotoxicity against human lung adenocarcinoma cells, regardless of their *p53* status, by inducing apoptosis associated with mitochondrial perturbation, and proposing the potential to employ *P. cocos* in the treatment of lung cancer.

## 1. Introduction

Lung cancer is known as the most prevalently diagnosed cancer worldwide [1]. Despite considerable advances in cancer diagnosis and management, the prognosis of lung cancer patients continues to be unsatisfactory, as the five-year survival rate is less than 16%. Thus, lung cancer predominantly accounts for the leading cause of cancer-associated death, and was reported to cause approximately 1.69 million deaths in 2015 [1,2].

In the last decade, mushrooms, especially medicinal ones, have been demonstrated to be promising and productive natural sources of bioactive compounds with various pharmacological properties that are effective for cancer management, such as cytotoxicity against cancer cells, as well as antioxidant, anti-angiogenic, and immunomodulatory activities [3,4]. Therefore, it is clinically significant to screen mushrooms with therapeutic potential for lung cancer intervention and elucidate the chemical and molecular basis for their anticancer activity. Indeed, protein-bound polysaccharide K (PSK) isolated from *Coriolus versicolor* was approved in Japan for its clinical use to treat patients with gastric, colorectal, and small-cell lung cancer [5]. In addition, various fungal metabolites and their derivatives, including aphidicolin, fumagillin, and phenylahistin, are currently being evaluated for their anticancer efficacy in clinical trials [6].

*Poria cocos* Wolf is a fungus that belongs to the Polyporaceae family, and is extensively distributed in East Asia, including Korea, China, and Japan, and can be commonly seen on the roots and dead bark of pine trees. In traditional East Asian medicine, this mushroom, especially the epidermis of its sclerotium (known as “Fu-Ling-Pi” in Chinese), has been broadly utilized for the treatment of various medical conditions, including insomnia, urinary dysfunction, and diarrhea [7].

Of note, polysaccharides and lanostane-type triterpenoids derived from the sclerotium and mycelium of *P. cocos*, respectively, exhibited antitumor activity in vivo in murine tumor models [8,9]. In addition to the potent antineoplastic properties, this mushroom has been demonstrated to exhibit anti-inflammatory, antioxidant, and immunomodulatory activities, as well as cytotoxicity against cancer cells [7,10]. Chemical and pharmacological studies on the sclerotium of *P. cocos* have identified lanostane-type triterpenoids and polysaccharides as two principal constituents that are responsible for its anticancer activity [7]. In particular, pachymic acid and β-d-glucan have been found to exert cytotoxicity by promoting apoptosis mediated by mitochondrial and/or death-receptor pathways in different types of human cancer cells, including breast cancer, leukemia, melanoma, pancreatic cancer, prostate cancer, and ovarian cancer cells [7,11,12,13]. Taken together, these previous findings strongly suggest the potential application of *P. cocos* and its bioactive compounds in the treatment of a wide range of cancer types.

However, only a few studies have reported the biological effects of *P. cocos* and its constituents on human lung cancer cells to date [12,14,15,16]. In addition, most of these studies only examined cancer cells harboring wild-type *p53*, which is a tumor suppressor [12,15,17]. Considering that *p53* has been found to be mutated in more than 50% of human cancers and is known to be responsible for chemoresistancy in cancer patients [18], the biological activities of *P. cocos* and its constituents need to be further evaluated in human lung cancer cells with various *p53* statuses so that the therapeutic potential of these components against lung cancer can be verified and broadened. Furthermore, little is known about the biological activities and the underlying molecular mechanisms of constituents of *P. cocos* other than lanostane-type triterpenoids and polysaccharides in human lung cancer cells.

In the current study, in order to continue with our efforts to screen mushrooms that manifest anticancer potential against lung cancer and identify compounds that contribute to the activity [19,20,21], we evaluated the biological activity of an EtOH extract of the sclerotia of *P. cocos* in four human lung adenocarcinoma cell lines, A549, H1264, H1299, and Calu-6, accompanying different *p53* status. We also chemically investigated the EtOH extract to identify the bioactive compounds responsible for its biological actions in lung cancer cells. We further explored the molecular mechanisms underlying the biological activities of the EtOH extract and the isolated compounds.

## 2. Materials and Methods

### 2.1. Cell Culture

Four human lung adenocarcinoma cell lines—A549, H1264, H1299, and Calu-6—were kindly provided by Dr. Steven M. Albelda (Perelman School of Medicine, University of Pennsylvania, Philadelphia, PA, USA) and cultured in RPMI-1640 medium (WelGENE, Seoul, Korea) supplemented with 10% fetal bovine serum (FBS, Gemini Bio-Products, West Sacramento, CA, USA), 2 mM of l-glutamine, 50 U/mL penicillin, and 50 μg/mL of streptomycin (WelGENE).

### 2.2. Cell Viability Analysis

A549, H1264, H1299 (5 × 10^3^ cells per well), and Calu-6 (7.5 × 10^3^ cells per well) cells were plated in triplicate in 96-well tissue culture plates (Thermo Scientific, Waltham, MA, USA) and grown overnight. Cells were then treated with the EtOH extract of the sclerotia of *P. cocos*, and the compounds isolated from it at various concentrations. Cells were also incubated in growth medium containing dimethyl sulfoxide (DMSO) at concentrations ranging from 0 to 0.5% and 0 to 1.25% as vehicle controls for the treatments with the EtOH extract and the isolated compounds, respectively. At 48 h after the treatment, cell viability was assessed through a WST-1 cell proliferation assay with an EZ-Cytox Enhanced Cell Viability Assay kit (Daeil Lab Service, Seoul, Korea) in accordance with the manufacturer’s instructions, as previously described [22]. Cell viability was determined as a percentage of that of the corresponding vehicle control. The IC_50_ values of the EtOH extract and the isolated compounds were estimated by a non-linear regression analysis of the dose-response curve in GraphPad Prism 5.0 (GraphPad Software, Inc., San Diego, CA, USA).

### 2.3. TUNEL Assay

A549, H1264, H1299 (7.5 × 10^3^ cells), and Calu-6 (1.0 × 10^4^ cells) were seeded in triplicate on 12-mm glass coverslips (Marienfeld GmbH, Lauda-Königshofen, Germany) and grown overnight. The cells were then treated with the EtOH extract of the sclerotia of *P. cocos* and the isolated compounds. Cells were also treated with growth medium containing DMSO as a vehicle control. After 48 h of treatment, apoptotic cells were detected by terminal deoxyribonucleotidyl transferase-mediated deoxyuridine triphosphate (dUTP) nick end labeling (TUNEL) staining with a Dead-End labeling kit (Promega, Madison, WI, USA) according to the manufacturer’s protocol, as previously described [22]. The cells were also counterstained with 0.5 μg/mL of 4′,6-diamidino-2-phenylindole (DAPI, Sigma, St. Louis, MO, USA) so that the nuclei could be visualized. The stained cells were then examined under a fluorescence microscope (Carl Zeiss, Jena, Germany), and the percentage of apoptotic cells was calculated as the ratio of TUNEL-positive cells to the total number of cell nuclei counted in six randomly selected high-power fields (400×) on each slide.

### 2.4. Immunoblotting

Calu-6 cells (1 × 10^6^) were plated on 100-mm tissue culture dishes (Thermo Scientific Instrument Co., Boston, MA, USA), grown overnight, and treated with the EtOH extract of the sclerotia of *P. cocos* and the isolated compounds. Cells treated with 1 μM of doxorubicin (Sigma) and DMSO were used as positive and vehicle controls, respectively. After treatment for 48 h, the cells were harvested and lysed in radio-immunoprecipitation assay (RIPA) buffer containing 10 mM of NaF, 1 mM of Na_3_VO_4_, 1 μM of dithiothreitol (DTT), 1 mM of phenylmethane sulfonyl fluoride (PMSF) (Sigma), and a protease inhibitor cocktail (Roche, Mannheim, Germany). The whole-cell lysates were then separated by SDS-PAGE, transferred to a polyvinylidene difluoride (PVDF) membrane (BioRad, Hercules, CA, USA), and probed for poly (ADP-ribose) polymerase (PARP), caspase-3, cleaved caspase-3 (Cell Signaling Technology, Danvers, MA, USA), Bax, Bcl-2 (Santa Cruz Biotechnology, Santa Cruz, CA, USA), and β-actin (as a loading control, Thermo Scientific), as previously described [22]. 

### 2.5. Statistical Analysis

Two-tailed unpaired Student’s *t* tests were employed to determine the statistical significance of differences between the treated and control groups. All of the data are presented as the mean ± standard error of the mean (SEM), and *p* values less than 0.05 were considered statistically significant.

## 3. Results

### 3.1. Cytotoxicity of the EtOH Extract of the Sclerotia of P. cocos toward Human Lung Adenocarcinoma Cells with Different p53 Statuses

**Figure 1:**
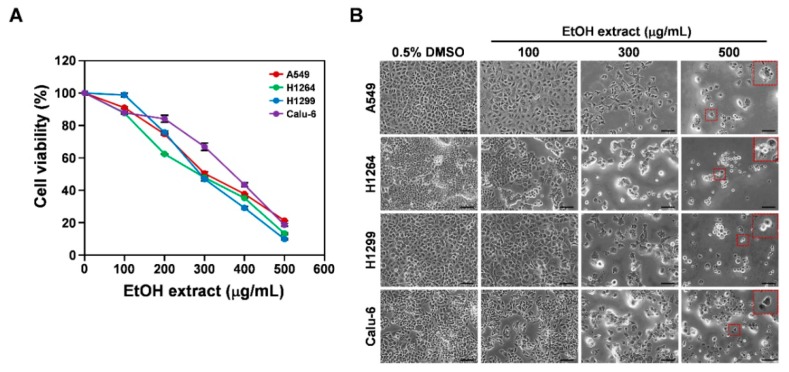
The EtOH extract of the sclerotia of *P. cocos* reduces cell viability in human lung adenocarcinoma cells, regardless of the *p53* status. (**A**) Cell viability assessed with the WST-1 assay in four human lung adenocarcinoma cell lines, A549, H1264, H1299, and Calu-6, 48 h after treatment with the EtOH extract at the indicated concentrations. (**B**) Representative bright-field images (200× total magnification) of human lung adenocarcinoma cells treated with the EtOH extract at the indicated concentrations for 48 h. Magnified regions are shown as insets. Data are presented as means ± SEMs. Scale bar: 100 μm.

The status of *p53* in cancer cells has been shown to correlate closely with their chemoresistance phenotype [18]. Therefore, in order to verify the previous findings on the cytotoxicity of *P. cocos* toward human lung cancer cells [17] and determine its relationship to the *p53* status of the cells, we prepared an EtOH extract of the sclerotia of *P. cocos*, and examined its effects on cell viability in four human lung adenocarcinoma cell lines accompanying different *p53* statuses: A549 (*p53*-wild-type), H1264 (*p53*-mutated), H1299 (*p53*-null), and Calu-6 (*p53*-null) [23,24] (Figure 1 and Table 1).

Consistent with the previous study [15], a WST-1 cell viability assay showed that the EtOH extract exhibited cytotoxicity toward A549 cells after 48 h of treatment in a dose-dependent manner, with an IC_50_ value of 301.1 μg/mL (Figure 1A and Table 1). In addition, the EtOH extract significantly and dose-dependently reduced cell viability in H1264, H1299, and Calu-6 cells, with IC_50_ values ranging from 284.0 μg/mL to 372.7 μg/mL (Figure 1A and Table 1). However, no notable correlation was examined between the cytotoxic effect of the EtOH extract and the *p53* status of the human lung cancer cells, implying the cytotoxicity of *P. cocos* in human lung cancer cells, irrespective of their *p53* status.

**Figure 2:**
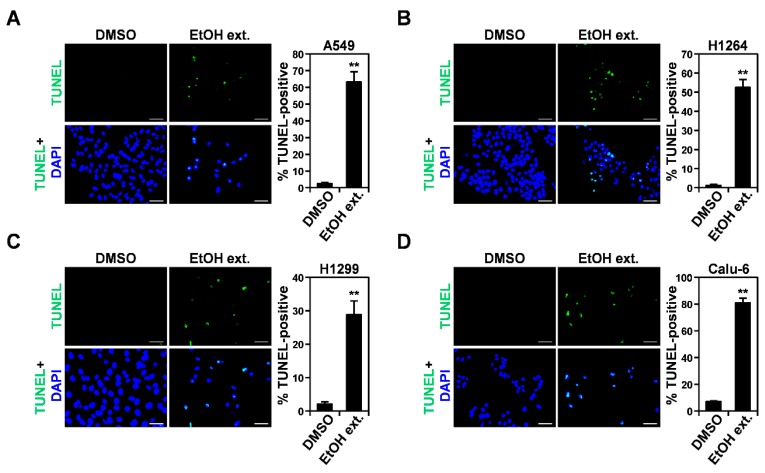
The EtOH extract of the sclerotia of *P. cocos* induces apoptosis in human lung adenocarcinoma cells. (**A**–**D**) Representative fluorescence images (400× total magnification) of TUNEL (green) and 4′,6-diamidino-2-phenylindole (DAPI) (blue) staining (**left**), and quantitation of TUNEL-positive cells (**right**), in A549 (**A**), H1264 (**B**), H1299 (**C**), and Calu-6 (**D**) cells treated with 400 μg/mL of the EtOH extract or 0.4% DMSO as a vehicle control for 48 h. Data are presented as means ± SEMs. Scale bar: 50 μm. ** p < 0.01.

*P. cocos* and its constituents, including lanostane-type triterpenoids and polysaccharides, have been demonstrated to promote apoptosis in a variety of human cancer cells [7,17]. In parallel with these previous findings, all of the human lung cancer cells tested in the current study were found to undergo morphological changes typical of apoptosis, which include cell rounding, cell shrinkage, cell membrane blebbing, and the detachment of the cell from the substratum [25], after treatment with the EtOH extract of the sclerotia of *P. cocos* (Figure 1B). This suggests that the cytotoxicity of the EtOH extract in the examined lung cancer cells is mediated by pro-apoptotic activities. To further confirm the promotion of apoptosis in human lung cancer cells, we performed TUNEL staining on A549, H1264, H1299, and Calu-6 cells that are treated with the EtOH extract for 48 h (Figure 2). As predicted, the proportion of TUNEL-positive cells was significantly greater following EtOH extract treatment than it was following DMSO treatment (the vehicle control) in all of the cancer cell lines tested (Figure 2A–D). These observations not only demonstrate that the cytotoxicity of the EtOH extract toward the cancer cells is attributable to its pro-apoptotic activity, but also suggest that apoptosis induced by the extract in the cancer cells is mediated by *p53*-independent pathways.

Collectively, our results reveal that *P. cocos* exerts cytotoxicity by inducing apoptosis in human lung cancer cells, regardless of the *p53* status of the cancer cells, and thus further confirms the potential of *P. cocos* to be applied in the treatment of lung cancer.

### 3.2. Chemical Analysis of Bioactive Compounds from the EtOH Extract of the Sclerotia of P. cocos

**Figure 3:**
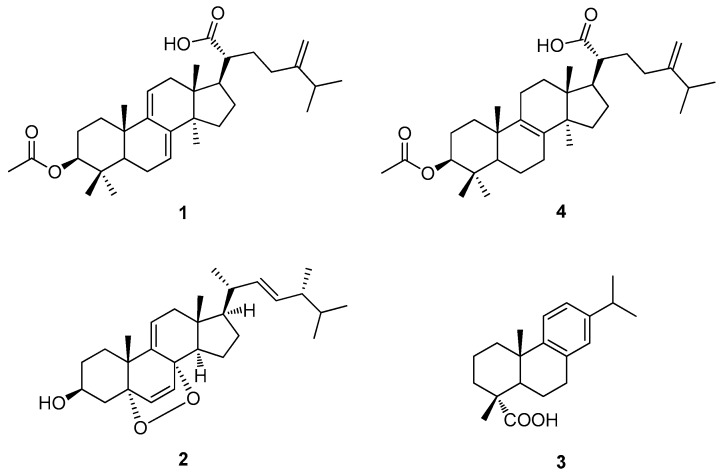
Structures of compounds (**1**–**4**) isolated from the sclerotia of *P. cocos*.

To determine the major components of the sclerotium of *P. cocos* contributable to its cytotoxicity against human lung cancer cells, the EtOH extract was fractionated into four different fractions (hexane-soluble, CH_2_Cl_2_-soluble, EtOAc-soluble, and *n*-BuOH-soluble fractions), and sequentially analyzed by LC/MS. The hexane-soluble fraction was found to contain the main constituents. This fraction was chemically investigated by repeated column chromatography and HPLC techniques (see Supplementary Materials), where two triterpenoids (**1** and **4**), a sterol (**2**), and a diterpenoid (**3**) were successfully isolated (Figure 3). Through comparison of spectroscopic and physical data of the isolates with those previously reported, along with LC/MS analysis, the triterpenoids were identified as dehydroeburicoic acid monoacetate (**1**) [26] and acetyl eburicoic acid (**4**) [27], the sterol was identified as 9,11-dehydroergosterol peroxide (**2**) [28], and the diterpenoid was identified as dehydroabietic acid (**3**) [29].

### 3.3. Cytotoxicity of Isolated Compounds ***1**–**4*** toward Human Lung Adenocarcinoma Cells

**Figure 4:**
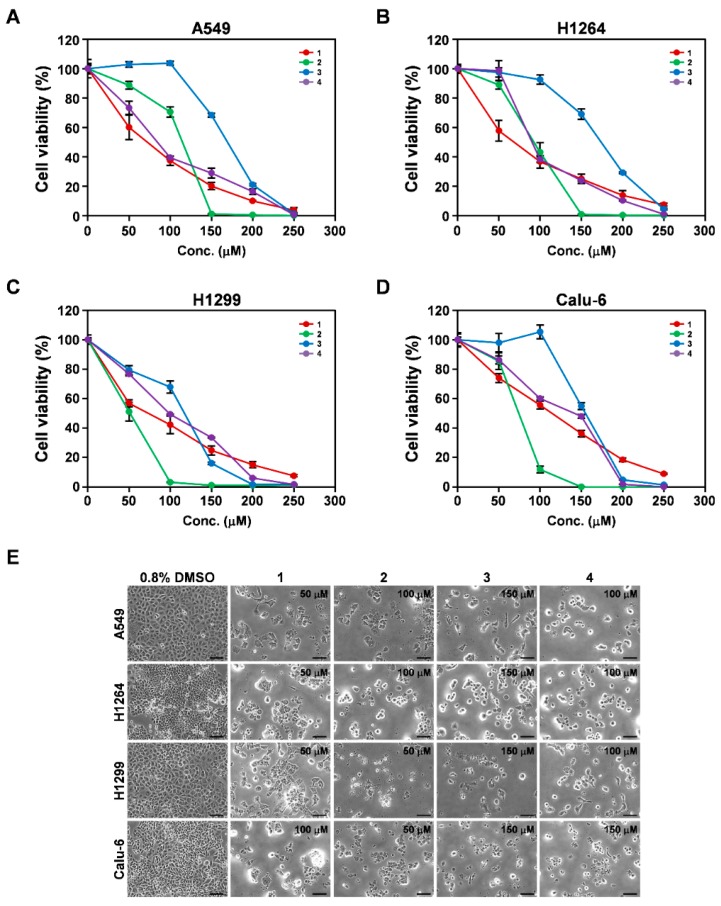
Cytotoxicity of compounds isolated from the EtOH extract of the sclerotia of *P. cocos* toward human lung adenocarcinoma cells. (**A**–**D**) Cell viability in A549 (**A**), H1264 (**B**), H1299 (**C**), and Calu-6 (**D**) cells, assessed with the WST-1 assay 48 h after treatment with the isolated compounds (**1**–**4**) at the indicated concentrations. (**E**) Representative bright-field images (200× total magnification) of human lung adenocarcinoma cells treated with the isolates at the indicated concentrations or 0.8% DMSO as a vehicle control. Data are presented as means ± SEMs. Scale bar: 100 μm.

Compounds **1** and **4** were previously identified in the sclerotia of *P. cocos* [30,31]. However, their biological effects in cancer cells, including lung cancer cells, have not yet been examined. In addition, to the best of our knowledge, compounds **2** and **3** were identified in *P. cocos* for the first time in this study. To determine whether these compounds, which were isolated as the main constituents of the EtOH extract of the sclerotia of *P. cocos*, contributed to the cytotoxicity of the extract against human lung cancer cells in vitro, we investigated their effects on the cell viability of the same human lung adenocarcinoma cell lines (Figure 4 and Table 1). WST-1 assay was carried out and revealed that after 48 h of treatment, compounds **1**, **2**, **3**, and **4** showed significant dose-dependent cytotoxicity toward all of the cancer cell lines tested, with IC_50_ values ranging from 63.6 to 108.0 μM, 50.6 to 121.9 μM, 122.5 to 171.0 μM, and 75.8 to 141.7 μM, respectively (Figure 4A–D and Table 1). In addition, their cytotoxic effects did not correlate notably with the *p53* status of the cancer cells. These observations imply that all of the isolated compounds contributed to the cytotoxicity of the EtOH extract of the sclerotia of *P. cocos* toward human lung cancer cell lines in vitro.

**Figure 5:**
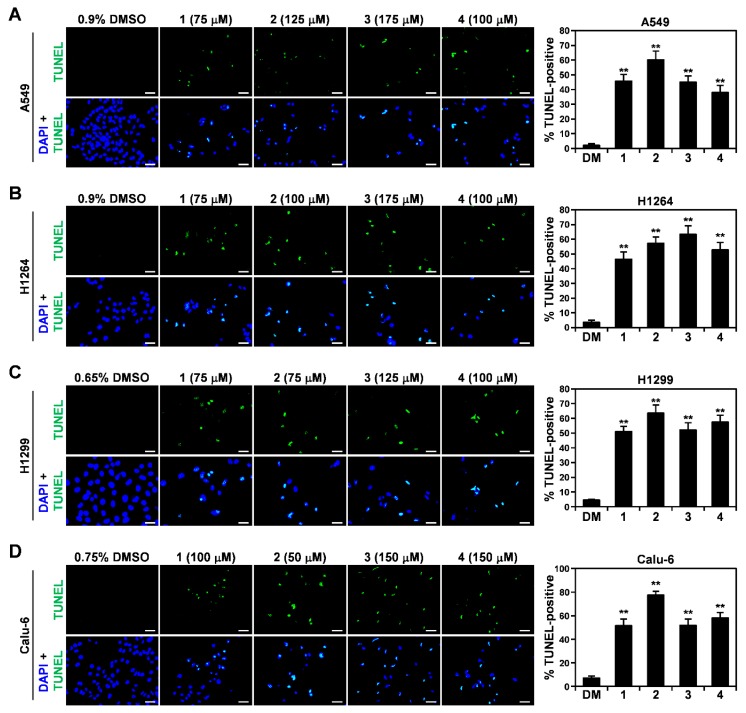
Pro-apoptotic effects of isolated compounds **1**–**4** in human lung adenocarcinoma cells. (**A**–**D**) Representative fluorescence images (400× total magnification) of TUNEL (green) and DAPI (blue) staining (**left**), and quantitation of TUNEL-positive cells (**right**), in A549 (**A**), H1264 (**B**), H1299 (**C**), and Calu-6 (**D**) cells treated with the compounds (**1**–**4**) or DMSO (DM) as a vehicle control at the indicated concentrations for 48 h. Data are presented as means ± SEMs. Scale bar: 50 μm. ** p < 0.01.

Similar to the EtOH extract, all four isolates were observed to increase the cell population exhibiting an apoptotic morphology in all of the human lung cancer cells tested (Figure 4E). To validate that the isolated compounds triggered apoptosis in the cancer cells, we performed TUNEL staining on A549, H1264, H1299, and Calu-6 cells treated with each isolated compound for 48 h (Figure 5). As predicted, all of the compounds showed a significant increase in the TUNEL-positive cell populations of all of the tested cancer cell lines, compared to DMSO (the vehicle control) (Figure 5A–D), demonstrating that all of the isolated compounds exerted cytotoxicity in human lung cancer cells through the promotion of apoptosis.

Taken together, these results support our notion that the isolated compounds **1**–**4** are the main bioactive components contributing to both the cytotoxic and pro-apoptotic effects of the EtOH extract of the sclerotia of *P. cocos* examined in human lung cancer cells in vitro.

### 3.4. Induction of the Mitochondrial Apoptotic Pathway by the EtOH Extract of the Sclerotia of P. cocos and Its Cytotoxic Constituents in Human Lung Adenocarcinoma Cells

**Figure 6:**
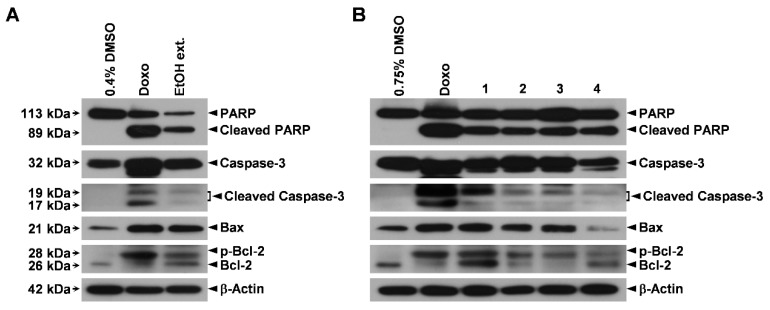
The EtOH extract of the sclerotia of *P. cocos* and isolated compounds **1**–**4** activate caspase-3 and alter Bax and Bcl-2 proteins in human lung adenocarcinoma cells. (**A**,**B**) Calu-6 cells were treated for 48 h with 400 μg/mL of the EtOH extract (**A**) and with 100 μM, 75 μM, 150 μM, and 150 μM of compounds **1**, **2**, **3**, and **4**, respectively (**B**). Cells treated with 1 μM of doxorubicin (Doxo) and 0.4 or 0.75% DMSO served as positive and vehicle controls, respectively. Whole-cell lysates were then prepared and probed for caspase-3, cleaved caspase-3, PARP, Bax, Bcl-2, and β-actin (a loading control).

To elucidate the molecular mechanisms underlying the pro-apoptotic activities of the EtOH extract of the sclerotia of *P. cocos* and its constituents in human lung cancer cells, we first explored their effects on the activation of caspase-3 (a major downstream effector caspase in the apoptotic pathway [32]) and the cleavage of its substrate PARP [33] in Calu-6 cells (Figure 6). Upon treatment of Calu-6 cells with the EtOH extract, a dramatic increase in the cleaved forms of both caspase-3 and PARP compared to their levels in cells treated with DMSO (Figure 6A) was observed. Furthermore, compounds **1**–**4** were all found to induce the cleavage of both caspase-3 and PARP in these cells (Figure 6B). These data suggest that the EtOH extract and the constituents isolated from it induced apoptosis in human lung cancer cells through a caspase-3-dependent mechanism.

It is well known that the upregulation of Bax and the inactivation of Bcl-2 (through downregulation or hyperphosphorylation) reduce the mitochondrial membrane potential, leading to caspase-dependent apoptosis [34,35]. Therefore, to further delineate the upstream pathway leading to caspase-3 activation by the EtOH extract and the isolated compounds, we performed an immunoblot assay to probe lysates of Calu-6 cells treated with the extract and compounds for Bax and Bcl-2 proteins (Figure 6). Calu-6 cells were also treated with doxorubicin, which is an anticancer drug known to increase Bax protein expression and induce Bcl-2 protein hyperphosphorylation [36,37], as a positive control. Of note, Bax protein expression and Bcl-2 protein phosphorylation were significantly greater in Calu-6 cells treated with the EtOH extract than in those treated with the vehicle (Figure 6A). In addition, compounds **1**, **2**, and **3** increased the protein levels of both Bax and hyperphosphorylated Bcl-2 in these cells compared to the vehicle control (Figure 6B). Although compound **4** did not upregulate Bax, it did increase the hyperphosphorylated form of Bcl-2 compared to DMSO treatment in Calu-6 cells (Figure 6B). These observations imply that the EtOH extract and isolated compounds **1**–**4** promoted apoptosis in human lung cancer cells by perturbing the mitochondria.

Correspondingly, our data strongly suggest that the sclerotia of *P. cocos* exert cytotoxicity against human lung cancer cells by the promotion of apoptosis mediated by the caspase-3-dependent mitochondrial pathway, and further support our notion that the isolated compounds, which are considered as the main constituents of the sclerotia of *P. cocos*, are responsible for the cytotoxicity.

## 4. Discussion

In the last 20 years, *P. cocos* and its constituents, especially triterpenoids such as pachymic acid and polysaccharides such as β-glucan, have been found to exhibit a broad spectrum of antitumor activities both in vitro and in vivo, demonstrating the potential use of *P. cocos* as a functional food and a natural source of novel lead compounds for cancer management [7]. However, the anticancer potential of this mushroom and its constituents against human lung cancer required further investigation. In addition, the biological activity of ingredients other than triterpenoids and polysaccharides remained poorly studied.

In this study, an EtOH extract of the sclerotia of *P. cocos* exhibited significant cytotoxicity toward human lung cancer cells in vitro. This cytotoxicity was found to be independent of the *p53* status of the cancer cells, and was mediated by the caspase-3-dependent mitochondrial apoptosis pathway. These observations not only support the results from previously published studies on the cytotoxicity of *P. cocos* toward A549 cells [12,15,17], they also provide further insight into the molecular mechanisms underlying this biological activity in human lung cancer cells. Considering that *p53*, a pivotal tumor suppressor gene, is mutated in more than 50% of human cancers, and that its status correlates closely with the clinical outcomes of cancer patients receiving chemotherapy and with the drug responsiveness of cancer cells in vitro [18], our results also broaden the potential application of *P. cocos* in lung cancer treatment.

Our chemical investigation of the sclerotia of *P. cocos* revealed two triterpenoids, dehydroeburicoic acid monoacetate (**1**) and acetyl eburicoic acid (**4**); a sterol, 9,11-dehydroergosterol peroxide (**2**); and a diterpenoid, dehydroabietic acid (**3**), as the main constituents. All four isolated compounds were found to exert cytotoxicity by inducing apoptosis along with the activation of caspase-3 in human lung cancer cells. Compounds **1**–**3** stimulated both the expression of a pro-apoptotic protein, Bax, and the hyperphosphorylation of an anti-apoptotic regulator, Bcl-2, in human lung cancer cells. Compound **4** did not upregulate the Bax protein, but it did increase the level of hyperphosphorylated Bcl-2 in these cells. As with increased Bax expression, Bcl-2 hyperphosphorylation perturbs the mitochondrial membrane integrity by interfering with the interaction between Bcl-2 and pro-apoptotic partners such as Bax, leading to caspase-3 activation [34,35,38]. Hence, our data suggest that all four isolated compounds induce apoptosis through the mitochondrial pathway, similar to the EtOH extract of the sclerotia of *P. cocos*, and thus are the main bioactive constituents responsible for the cytotoxicity of *P. cocos* toward human lung cancer cells.

Triterpenoids derived from natural sources including mushrooms are well known for their anticancer potential, as they exert antioxidant, anti-inflammatory, and immunomodulatory activities and cytotoxicity against a broad spectrum of cancer cells [10,39]. *P. cocos* is a plentiful source of triterpenoid derivatives [7], suggesting its potential application in cancer prevention and treatment. So far, more than 50 triterpenoid derivatives have been identified from *P. cocos*, and their biological activities toward various cancer types have been evaluated in vitro and in vivo [7]. In this study, we identified two triterpenoid derivatives, dehydroeburicoic acid monoacetate (**1**) and acetyl eburicoic acid (**4**), as the main constituents of the sclerotia of *P. cocos* with cytotoxicity toward human lung cancer cells. Dehydroeburicoic acid monoacetate (**1**), which was identified in *P. cocos* for the first time in our previous study [30], had not previously been examined for its biological activity in cancer cells, and the present study is the first to demonstrate its cytotoxicity against cancer cells and the underlying mechanism. Acetyl eburicoic acid isolated from *Laetiporus sulphureus* was previously shown to exert cytotoxicity against HL-60 cells, which is a human myeloid leukemia cell line, by inducing caspase-3-dependent apoptosis [27]. In parallel with this finding, we found that acetyl eburicoic acid (**4**) derived from *P. cocos* exerted cytotoxic effects by triggering apoptosis accompanied by caspase-3 activation and mitochondrial destabilization in human lung cancer cells. Taken together, our findings provide additional experimental evidence for the anticancer potential of triterpenoid derivatives isolated from *P. cocos*, and thus further support the potential application of *P. cocos* for the treatment of lung cancer in particular.

Dehydroabietic acid (a naturally occurring diterpene rosin acid) and its synthetic derivatives have exhibited cytotoxicity toward various types of human cancer cells, including lung cancer cells [40,41]. Although the methyl ester derivative of dehydroabietic acid was previously identified in *P. cocos* and was shown to inhibit the tumor-promoting activity of 12-*O*-tetradecanoylphorbol-13-acetate (TPA) in vitro [42], dehydroabietic acid (**3**) was isolated from *P. cocos* and evaluated for its biological effects on human lung cancer cells for the first time in the present study. In addition, compound **2**, a sterol identified as 9,11-dehydroergosterol peroxide, was isolated from *P. cocos* for the first time in this study. Although 9,11-dehydroergosterol peroxide isolated from the mycelium of *Ganoderma lucidum* has been evaluated for its cytotoxicity against human hepatocellular carcinoma and melanoma cells [28,43], its biological effects have not been examined in lung cancer cells. In the present study, we demonstrated that 9,11-dehydroergosterol peroxide exerts cytotoxicity against human cancer cells by inducing apoptosis. This finding is supported by previous reports that the apoptosis induced by this sterol was caspase-dependent and mediated through the mitochondrial pathway [43]. Taken together, our findings indicate that constituents of *P. cocos* other than triterpenoids and polysaccharides also contribute to its in vitro cytotoxic activity against human cancer cells, especially lung cancer cells.

Overall, we demonstrated that the cytotoxicity of *P. cocos* against human lung cancer cells with different *p53* statuses in vitro and elucidated the underlying chemical and molecular basis for this activity. Although the antitumor activity of *P. cocos* needs to be validated in vivo in lung tumor models, our findings suggest the potential application of *P. cocos* and the isolated compounds (**1**–**4**) in lung cancer therapy.

## 5. Conclusions

Our study demonstrates that *P. cocos* and its four main bioactive compounds, including two triterpenoids (**1** and **4**), a sterol (**2**), and a diterpenoid (**3**), are cytotoxic to human lung cancer cells with different *p53* statuses. In terms of the molecular mechanism, our study indicates that *P. cocos* and these isolated compounds exert cytotoxicity against human lung cancer cells by inducing apoptosis accompanied by caspase-3 activation and mitochondrial perturbation. These findings provide experimental evidence and a molecular explanation for the anticancer properties of *P. cocos*, especially against lung cancer, and support the previous notion that *P. cocos* can be applied for cancer treatment.

## Figures and Tables

**Table 1:** IC_50_ values of the EtOH extract of the sclerotia of *P. cocos* and the isolated compounds in human lung cancer cell lines.

Cell Lines	EtOH Ext. (μg/mL)	Compounds (μM)			
		1	2	3	4
A549	301.1 ± 7.2 ^1^	64.4 ± 13.0	121.9 ± 5.2	163.8 ± 1.7	75.8 ± 3.8
H1264	284.0 ± 3.6	63.6 ± 12.3	92.3 ± 7.9	171.0 ± 4.1	82.5 ± 4.6
H1299	285.5 ± 4.9	66.6 ± 2.5	50.6 ± 6.7	122.5 ± 3.5	96.8 ± 0.8
Calu-6	372.7 ± 2.0	108.0 ± 8.5	71.2 ± 3.4	154.5 ± 2.2	141.7 ± 6.0

^1^ Values are the mean ± SEM of triplicate determinations.

## Supplementary Materials

The supplementary materials are available online http://www.mdpi.com/2073-4409/7/9/116/s1.
