# Supplementary material for: Cytotoxic Constituents from the Sclerotia of Poria cocos against Human Lung Adenocarcinoma Cells by Inducing Mitochondrial Apoptosis
Source: Cells. 2018 Aug 24;7(9):116. doi: 10.3390/cells7090116 (PMC6162800; doi:10.3390/cells7090116)
Supplement: Supplementary file 1 [file cells-07-00116-s001.pdf]

Supplementary Information

Cytotoxic constituents from the sclerotia of *Poria cocos* against human lung adenocarcinoma cells by inducing mitochondrial apoptosis

Seulah Lee [1,a], Seul Lee [2,a], Hyun-Soo Roh [2], Seong-Soo Song [2], Rhim Ryoo [3], Changhyun Pang [4], Kwan-Hyuck Baek [2,*] and Ki Hyun Kim [1,*]

[1] School of Pharmacy, Sungkyunkwan University, Suwon 16419, Republic of Korea; sarahlee0801@gmail.com (S.L.)

[2] Department of Molecular and Cellular Biology, Samsung Medical Center, Sungkyunkwan University School of Medicine, Suwon 16419, Republic of Korea; leeseul6988@naver.com (S.L.); hyunsno@naver.com (H.-S.R.); songll91@naver.com (S.-S.S.)

[3] Special Forest Products Division, Forest Bioresources Department, National Institute of Forest Science, Suwon 16631, Republic of Korea; rryoo@korea.kr (R.R.)

[4] School of Chemical Engineering, Sungkyunkwan University, Suwon 16419, Republic of Korea; chpang@skku.edu (C.P.)

[a] These authors contributed equally to this work

* Correspondence: khbaek@skku.edu (K.-H.B.); Tel.: +82-31-299-6162; khkim83@skku.edu (K.H.K.); Tel.: +82-31-290-7700

2.1. General experimental procedures

Infrared (IR) spectra were recorded on a Bruker IFS-66/S FT-IR spectrometer. Ultraviolet (UV) spectra were recorded with an Agilent 8453 UV-visible spectrophotometer (Agilent Technologies, CA, USA). Nuclear magnetic resonance (NMR) spectra were obtained from a Bruker AVANCE III 700 NMR spectrometer operating at 700 MHz (^{1}H) and 175 MHz (^{13}C) (Bruker), with chemical shifts given in ppm (δ). LC/MS analysis was performed on an Agilent 1200 Series HPLC system equipped with a diode array detector, a 6130 Series ESI mass spectrometer, and an analytical Kinetex C18 100 Å column (100 mm × 2.1 mm i.d., 5 μm) (Phenomenex, Torrance, CA, USA). Preparative high-performance liquid chromatography (HPLC) was performed with a Waters 1525 Binary HPLC pump with a Waters 996 Photodiode Array (PDA) Detector (Waters Corporation, Milford, CT, USA). Semi-preparative HPLC was conducted with a Shimadzu Prominence HPLC System with SPD-20A/20AV Series Prominence HPLC UV-Vis Detectors (Shimadzu, Tokyo, Japan). Silica gel 60 (Merck, 230-400 mesh) and reversed-phase (RP)-C_{18} silica gel (Merck, 230-400 mesh) were used for column chromatography. Merck precoated silica gel F_{254} plates and RP-18 F_{254s} plates were used for thin-layer chromatography (TLC). Spots on the TLC plates were detected under UV light or by heating after spraying with anisaldehyde-sulfuric acid.

2.2. Sample material

Sclerotia of *P. cocos* imported from China were purchased from Kyung-dong Herbal Medicine Market, Seoul, in January 2014. A voucher specimen of the material (PC1308) was identified by one of the authors (K. H. Kim) and deposited in the herbarium of the School of Pharmacy, Sungkyunkwan University, Suwon, Korea.

2.3. Extraction and isolation

Dried sclerotia of *P. cocos* (350.0 g) were extracted with 70% aqueous EtOH (each 2.0 L × 3 days) at room temperature. The extract was evaporated under reduced pressure with a rotavapor to yield the EtOH extract (23.3 g), which was suspended in distilled water (1.8 L) and MeOH (30.0 mL) and successively partitioned with hexane, dichloromethane (CH_2Cl_2), ethyl acetate (EtOAc), and *n*-butanol (BuOH). Four layers with increasing polarity were obtained: the hexane-soluble (6.5 g), CH_2Cl_2-soluble (2.3 g), EtOAc-soluble (2.7 g), and BuOH-soluble fractions (13.7 g). The hexane-soluble fraction (6.5 g) was separated by silica gel column chromatography (200 g, eluted with hexane/EtOAc [2:1]) to afford four fractions (H1-H4). Fraction H1 (4.3 g) was separated on an RP-C_{18} column eluted with 95% MeOH, yielding 8 subfractions (H11-H18). Subfraction H17 (112.0 mg) was

separated by semi-preparative HPLC (87% MeOH) on a Phenomenex Luna HPLC phenyl-hexyl column (250 × 10 mm; flow rate: 2 mL/min), yielding compound **4** (t_R 63.0 min, 1.7 mg). Fraction H2 (1.8 g) was chromatographed on an RP-C_{18} column (70 g, eluted with 90% MeOH), generating 7 subfractions (H21-H27). Subfraction H26 (46.0 mg) was fractionated by preparative HPLC (95% MeOH) on an Agilent Eclipse C18 column (21.2 × 250 mm; flow rate: 5 mL/min), and 9 subfractions (H261-269) were obtained. Subfraction H265 (10.3 mg) was purified by semi-preparative HPLC (92% MeOH) with a Phenomenex Luna phenyl-hexyl column (250 × 10 mm; flow rate: 2 mL/min) to afford compound **2** (t_R 20.5 min, 1.8 mg). On the same column, subfraction H268 (7.0 mg) was also separated by semi-preparative HPLC (87% MeCN), yielding compound **1** (t_R 18.0 min, 1.0 mg). Fraction H3 (585.0 mg) was subjected to RP-C_{18} column chromatography (25 g, eluted with 92% MeOH) to afford 5 subfractions (H31-35). Subfraction H33 (50.3 mg) was separated by semi-preparative HPLC (83% MeOH) with a Phenomenex Luna phenyl-hexyl column (250 × 10 mm; flow rate: 2 mL/min) to yield compound **3** (t_R 32.5 min, 3.9 mg).

The EtOH extract of the sclerotia of *P. cocos* and the compounds isolated from it were prepared as stock solutions at concentrations of 100 mg/mL and 20 mM, respectively, in dimethyl sulfoxide (DMSO, Sigma, St. Louis, MO, USA) and stored at -80°C until use.
